# Economic Burden of Pancreatic Cancer in Europe: a Literature Review

**DOI:** 10.1007/s12029-022-00821-3

**Published:** 2022-04-26

**Authors:** Diego Hernandez, Fabienne Wagner, Karla Hernandez-Villafuerte, Michael Schlander

**Affiliations:** 1 Division of Health Economics, German Cancer Research Center (DKFZ), Heidelberg, Germany; 2 Medical Faculty Mannheim, Heidelberg University, Mannheim, Germany

**Keywords:** Pancreas, Cancer, Economic Burden, Review, Europe

## Abstract

**Purpose:**

Pancreatic cancer is characterized by its high mortality, usually attributed to its diagnosis in already advanced stages. This article aims at presenting an overview of the economic burden of pancreatic cancer in Europe.

**Methods:**

A systematic literature review was conducted. It made use of the search engines EconLit, Google Scholar, PubMed and Web of Science, and retrieved articles published after December 31st, 1992, and before April 1st, 2020. Study characteristics and cost information were extracted. Cost per patient and cost per patient per month (PPM) were calculated, and drivers of estimate heterogeneity was analysed. Results were converted into 2019 Euros.

**Results:**

The literature review yielded 26 studies on the economic burden attributable to pancreatic cancer in Europe. Cost per patient was on average 40,357 euros (median 15,991), while figures PPM were on average 3,656 euros (median 1,536). Indirect costs were found to be on average 154,257 euros per patient or 14,568 euros PPM, while direct costs 20,108 euros per patient and 2,004 euros PPM. Nevertheless, variation on cost estimations was large and driven by study methodology, patient sample characteristics, such as type of tumour and cancer stage and cost components included in analyses, such as type of procedure.

**Conclusion:**

Pancreatic cancer direct costs PPM are in the upper bound relative to other cancer types; however, direct per patient costs are likely to be lower because of shorter survival. Indirect costs are substantial, mainly attributed to high mortality.

## Introduction

Pancreatic cancer occurs from malignant neoplasms originating from either the endocrine or the exocrine tissue of the pancreas, the latter component being the location of 95% of such tumours [1]. In 2018, around 460,000 individuals were diagnosed with this type of cancer worldwide and nearly as many died as a result of this disease [2, 3]. This high mortality rate is largely due to this type of cancer often being diagnosed in advanced stages. Patients in early stages show almost no symptoms, and once the symptoms appear, they are similar to those of many other diseases [4, 5]. Unfortunately, little progress has been made in treatment efficacy, leading to low survival rates. For example, 5-year survival rates were 10% in the USA from 2009 to 2015 and 9.5% in Germany from 2013 to 2015 [6–8]. Incidence is particularly pronounced in high-income countries, as the most common risk factors are older age, obesity and high plasma glucose [9]. For Europe, Ferlay, Partensky [10] forecasted that the number of deaths due to pancreatic cancer will increase at a rate of 50% between 2010 and 2025, making it the third most common cause of death from cancer by 2025.

Furthermore, this epidemiological burden is coupled with one of rising economic hardship attributable to the disease. In 2009, direct medical costs due to cancer corresponded to 4% of healthcare expenditures in Europe, while this figure represented 6.2% in 2018 [11, 12]. In Germany alone, direct costs from pancreatic cancer, excluding out of pocket payments, amounted about 721 million euros in 2015 [13]. Balancing economic burden with healthcare need is required in order to guarantee efficient resource allocation decisions [14, 15]. Nevertheless, to our knowledge, literature exploring cost estimations of pancreatic cancer in Europe is scarce, in particular at the patient level, limiting the measure of the extent of the economic burden.

The aim of this study is to provide estimations on the economic burden of pancreatic cancer in Europe, specifically, to present comprehensive information on direct and indirect costs at the patient level from the existing literature and thereby to identify possible drivers of heterogeneity in study figures. Carrato et al. [16] conducted a review on this topic in 2013, yielding only five studies. However, the number of articles including information on costs and pancreatic cancer has rapidly increased in recent years [17]. We seek to provide updated information on the current state of knowledge concerning the cost of pancreatic cancer, as well as an in-depth analysis of the estimates obtained.

## Methods

A literature review was completed following the Preferred Reporting Items for Systematic Reviews and Meta-Analyses (PRISMA) statements procedure [18]. The search engines employed were EconLit, Google Scholar, PubMed and Web of Science. Prior to the systematic literature research, a pilot search using the defined search terms was conducted in each of the four search engines in order to improve the search criteria. Search terms were defined by key words and synonyms describing the disease as well as its potential economic burden. The exact string of key terms employed in each search engine can be found in Appendix 1. The time horizon was restricted to articles published since 1993.

We initially followed the inclusion and exclusion criteria in Carrato et al. [16]. The criteria were adjusted based on the results of the pilot search. The updated criteria takes into account the following categories: study type, language of the article, publication date, data type, research subjects and content. Included were all articles published in peer review journals that are economic evaluations or interventional studies, in English language, after December 31st, 1992, until April 1st, 2020, which did research on humans. Any form of pancreatic cancer were included, and those which do not derive from the pancreas as primary site were excluded [19]. Studies that were included reported cost estimations for either one or more European countries or for Europe as a whole. Europe was defined as the countries in the European Union plus Iceland, Norway, Switzerland and the UK [20].

The title, abstract and full text screening were done by two persons independently (DH and FW). The reference lists of overviews, systematic reviews and meta-analyses found were further explored after title selection. Studies were excluded when at least one of the inclusion criteria was not met or at least one of the exclusion criteria was met. Disagreement reasons were stipulated and discussed between the two reviewers until agreement was reached.

Information extracted from the articles included study and patient sample characteristics, as well as cost estimations and the components included in these. Costs were extracted as average costs per patient and, if given, for subpopulations. For instance, whenever costs for a specific treatment were given, they were extracted as average cost per patient with that particular intervention. No incremental cost-effectiveness ratios were extracted. The quality of the studies to be included was evaluated using the checklist provided by Drummond and Jefferson [21] for partial and full economic evaluations and by Bennett and Manuel [22] for modelling studies. The data extraction and the quality assessment were done by a person (FW).

When the study provided cost per patient information for a number of subpopulations only (by treatment or cancer stage, for example), the average across the different subpopulations was calculated to obtain a cost per patient figure for that study. If the number of patients per subpopulation was available, the weighted average was calculated instead. Additionally, when possible, cost per patient estimations were transformed into cost per patient per month (PPM), to allow for cross study comparison. As the median survival in pancreatic cancer is less than 1 year, this metric is the most appropriate [23]. Some studies provided cost PPM explicitly; however, most of them did not. In the latter case, and if information on patient survival was available, cost PPM was calculated by dividing the average cost per patient by the average survival per patient in months. Cost values obtained were converted into 2019 euros using purchasing power parities (PPP) and the harmonized consumer price index (CPI) of the European Union [24, 25].

## Results

**Fig. 1:**
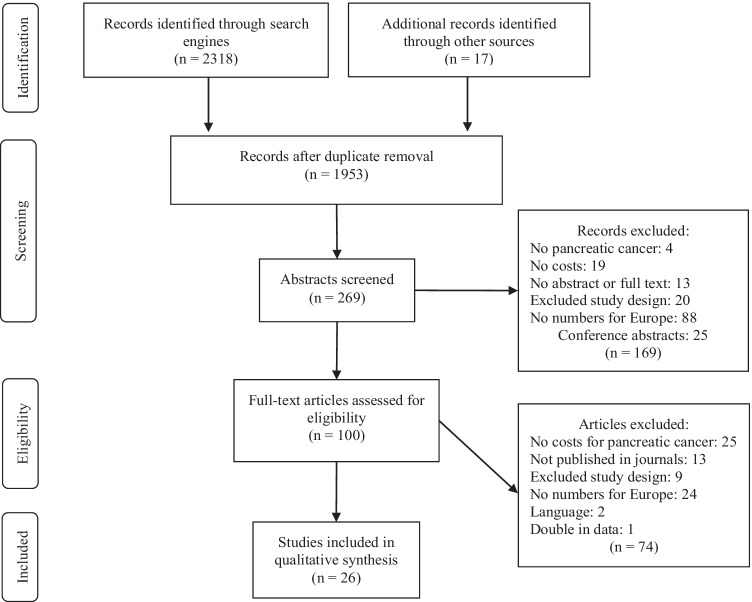
PRISMA flow chart for article selection

The literature review yielded 2,318 hits in total. In the end, a total of 26 studies met the inclusion criteria [26–51]. Figure 1 presents a flow chart showing the number of studies included and excluded at each step of the study selection process according to the PRISMA statement.

Table 1 presents an overview on the studies, as well as on the sample characteristics. From the selected 26 studies, 16 were published in 2013 or later [36–51]. All studies but one [41] focused on a single country. With eight studies each, the countries for which costs were assessed most frequently were the UK [28, 29, 40, 41, 43, 45, 46, 48] and Sweden [26, 30, 33, 34, 36, 37, 41, 44], followed by Italy with five [27, 39, 41, 47, 50], and Germany with three [32, 41, 51]. Cost-effectiveness analyses was the most common methodology, completed in 11 studies [26, 28, 29, 39, 40, 42, 44, 46–48, 51]. Also frequent were cost of illness analyses in eight studies [27, 30, 32, 34, 36, 45, 49, 50] and cost-utility analyses in seven studies [33, 35, 37, 38, 40, 43, 46]. Less often used were cost–benefit and burden of disease analyses, found in two [31, 49] and one study [41], respectively. Please note that a single study can consist of multiple methodologies. Moreover, 10 studies were based on modelling techniques [35, 38–40, 43–48], and the remaining 16 were observational studies.

**Table 1:** Study and sample characteristics of article selection

Author	Year	Country	Methodology	Perspective	Type of costs	Topic	Patient sample characteristics			
							Cancer type	Cancer stage	Age	Time horizon
Ragnarson-Tennvall and Wilking [26]	1999	Sweden	Cost-effectiveness	Healthcare sector	Direct medical, direct non-medical	Palliation and chemotherapy comparison	Pancreatic cancer	-	71 (mean)	Overall survival
Pasquali et al. [27]	2002	Italy	Cost of illness	Payer	Direct medical	Costing	Pancreatic cancer	All stages	72.1 (mean)	5 years
Aristides et al. [28]	2003	UK	Cost-effectiveness	Payer	Direct medical	Chemotherapy regimens comparison	Pancreatic cancer	II, III and IV	61/62 (median)	18 months
Bachmann et al. [29]	2003	UK	Cost-effectiveness	Service provider	Direct medical	Costs, hospital volume and doctor specialization	Pancreatic cancer	-	-	1 year
Hjelmgren et al. [30]	2003	Sweden	Cost of illness	Healthcare sector	Direct medical, direct non-medical	Costing	Pancreatic cancer	All stages	66.4 (mean)	Overall survival
Heinrich et al. [31]	2005	Switzerland	Cost–benefit	Service provider	Direct medical	Diagnostics and management strategy	Pancreatic cancer	All stages	61 (median)	18 months
Müller-Nordhorn et al. [32]	2005	Germany	Cost of illness	Payer, healthcare sector, society	Direct medical, direct non-medical, indirect	Costing	Pancreatic cancer	All stages	62 (mean)	2.5 years
Ljungman et al. [33]	2011	Sweden	Cost-utility	Service provider	Direct medical	Surgery	Exocrine/ampullary pancreatic adenocarcinoma	All stages	68 (mean)	Overall survival
Tingstedt et al. [34]	2011	Sweden	Cost of illness	Society	Direct medical, indirect	Costing	Pancreatic cancer excluding endocrine cancer	All stages	74 (median)	Overall survival
Walczak et al. [35]	2012	Poland	Cost-utility (Markov model)	Patient, payer	Direct medical	Chemotherapy regimens comparison	Pancreatic neuroendocrine tumours	All stages	-	Overall survival
Ansari et al. [36]	2013	Sweden	Cost of illness	Society	Direct medical, indirect	Chemotherapy regimens comparison	Pancreatic ductal adenocarcinoma	All stages	69 (median)	3 years
Ljungman et al. [37]	2013	Sweden	Cost-utility	Service provider	Direct medical	Palliation regimens comparison	Exocrine/ampullary pancreatic adenocarcinoma	-	66/69 (mean)	Overall survival
Carrato et al. [38]	2015	Spain	Cost-utility (Markov model)	Payer	Direct medical	Chemotherapy regimens comparison	Pancreatic adenocarcinoma	IV	63 (median)	10 years
Cucchetti et al. [39]	2015	Italy	Cost-effectiveness (Markov model)	Service provider	Direct medical	Surgery timing	Non-functioning pancreatic endocrine tumours	All stages	52 (median)	Overall survival
Gharaibeh et al. [40]	2015	UK	Cost-effectiveness, cost-utility (Markov model)	Payer	Direct medical	Chemotherapy regimens comparison	Pancreatic adenocarcinoma	IV	63 (median)	Overall survival
Hanly et al. [41]	2015	EU, Iceland	Burden of disease	Society	Indirect	Productivity loss	Pancreatic cancer	All stages	< 65	1 year
Joergensen et al. [42]	2016	Denmark	Cost-effectiveness	Service provider	Direct medical	High risk population screening	Pancreatic cancer	-	49 (median)	8 years
Gurusamy et al. [43]	2017	UK	Cost-utility (decision tree model)	Payer	Direct medical	Chemotherapy regimens comparison	Pancreatic cancer	-	-	5 years
Aronsson et al. [44]	2018	Sweden	Cost-effectiveness (Markov model)	Service provider	Direct medical	Management strategies comparison	Branch duct intraductal papillary mucinous neoplasm	-	65 (mean)	35 years
Briggs et al. [45]	2018	UK	Cost of illness (simulation model)	Payer	Direct medical	Costing	Pancreatic cancer	-	-	1 year
Gharaibeh et al. [46]	2018	UK	Cost-effectiveness, cost-utility (Markov model)	Payer	Direct medical	Chemotherapy regimens comparison	Ductal pancreatic adenocarcinoma	IV	> 60	Overall survival
Lazzaro et al. [47]	2018	Italy	Cost-effectiveness (Markov model)	Payer	Direct medical	Chemotherapy regimens comparison	Pancreatic cancer	IV	62/63 (median)	4 years
Mujica-Mota et al. [48]	2018	UK	Cost-effectiveness (semi Markov model)	Payer	Direct medical	Chemotherapy regimens comparison	Pancreatic neuroendocrine tumours	-	60 (mean)	40 years
Ahola et al. [49]	2019	Finland	Cost of illness, cost–benefit	Service provider	Direct medical	Hospital volume and resections	Pancreatic cancer	-	67 (median)	90 days
Morelli et al. [50]	2019	Italy	Cost of illness	Service provider	Direct medical	Diagnostics in surveillance follow-up	Pancreatic cyst neoplasm	-	67 (mean)	6 years
Thronicke et al. [51]	2020	Germany	Cost-effectiveness	Service provider	Direct medical	Integrative therapy	Pancreatic cancer	IV	63.7/68.6 (mean)	Overall survival

Study perspectives were those of the payers in 11 studies [27, 28, 32, 35, 38, 40, 43, 45–48], of the service provider in 10 studies [29, 31, 33, 37, 39, 42, 44, 49–51], of society in 4 studies [32, 34, 36, 41], of the healthcare sector in 3 studies [26, 30, 32] and of the patient in 1 study [35]. Two studies presented cost estimations for more than one perspective [32, 35]. Except for two studies, all provided figures for direct medical costs. One of these calculated the cost of a screening program [42], while the other one only productivity losses due to mortality for each of the countries in the European Union and Iceland [41]. Further, studies addressing costs other than direct medical were conducted for Germany or Sweden. Three studies included direct non-medical costs in their figures [26, 30, 32], which consisted, however, in transportation costs only. No additional components, such as the cost of informal care, were incorporated in these studies. Four studies estimated indirect costs, all of them assessed productivity losses: as a result of morbidity [36], mortality [41], or both causes [32, 34].

Table 2 presents in detail the cost components included in each study. As observed, costs associated with surgery were addressed in 14 studies [26, 27, 29–34, 37, 39, 43, 44, 49, 51], chemotherapy in 16 studies, [27–30, 32–38, 40, 46–48, 51] and palliative care in 8 studies [30, 35, 37, 38, 43, 44, 47, 48]. Most studies focusing on chemotherapy only are based on modelling techniques. Nevertheless, nearly half of the studies reported their estimations aggregately as total costs instead of breaking down figures according to single cost components or treatments. For example, costs for surgery are reported separately only in seven studies [27, 30, 34, 37, 39, 43, 44], for chemotherapy in seven studies [27, 28, 35, 38, 40, 47, 48] and for palliative care in five studies [35, 37, 39, 44, 48]. Furthermore, only two studies, both of them for Sweden, specify costs per cancer stage distinctly [30, 34].

**Table 2:** Cost components included in article selection

Author	Screening	Direct medical costs									Direct non-medical costs		Indirect costs	
		Diagnostics	Procedure				Hospitalization	Monitoring	Palliative care	Others	Transportation	Caregiver	Productivity loss	
			Surgery	Chemotherapy	Radiotherapy	Other							Mortality	Morbidity
Ragnarson-Tennvall and Wilking [26]	No	No	Yes	No	No	Yes	No	No	No	Yes	Yes	No	-	-
Pasquali et al. [27]	No	No	Yes	Yes	No	No	Yes	No	No	No	-	-	-	-
Aristides et al. [28]	No	No	No	Yes	No	Yes	Yes	No	No	Yes	-	-	-	-
Bachmann et al. [29]	No	Yes	Yes	Yes	Yes	Yes	Yes	No	No	Yes	-	-	-	-
Hjelmgren et al. [30]	No	Yes	Yes	Yes	Yes	Yes	Yes	No	Yes	No	Yes	No	-	-
Heinrich et al. [31]	No	Yes	Yes	No	No	No	No	No	No	No	-	-	-	-
Müller-Nordhorn et al. [32]	No	Yes	Yes	Yes	Yes	Yes	Yes	No	No	No	Yes	No	Yes	Yes
Ljungman et al. [33]	No	Yes	Yes	Yes	No	Yes	Yes	No	No	No	-	-	-	-
Tingstedt et al. [34]	No	Yes	Yes	Yes	No	Yes	Yes	No	No	No	-	-	Yes	Yes
Walczak et al. [35]	No	Yes	No	Yes	No	Yes	No	Yes	Yes	No	-	-	-	-
Ansari et al. [36]	No	Yes	No	Yes	No	Yes	Yes	No	No	No	-	-	No	Yes
Ljungman et al. [37]	No	Yes	Yes	Yes	No	Yes	Yes	No	Yes	No	-	-	-	-
Carrato et al. [38]	No	Yes	No	Yes	No	No	No	Yes	Yes	Yes	-	-	-	-
Cucchetti et al. [39]	No	Yes	Yes	No	No	Yes	No	Yes	No	No	-	-	-	-
Gharaibeh et al. [40]	No	Yes	No	Yes	No	Yes	No	Yes	No	No	-	-	-	-
Hanly et al. [41]	-	-	-	-	-	-	-	-	-	-	-	-	Yes	No
Joergensen et al. [42]	Yes	-	-	-	-	-	-	-	-	-	-	-	-	-
Gurusamy et al. [43]	No	No	Yes	No	No	No	Yes	Yes	Yes	No	-	-	-	-
Aronsson et al. [44]	No	Yes	Yes	No	No	Yes	Yes	Yes	Yes	No	-	-	-	-
Briggs et al. [45]	-	-	-	-	-	-	-	-	-	-	-	-	-	-
Gharaibeh et al. [46]	No	Yes	No	Yes	No	Yes	No	Yes	No	No	-	-	-	-
Lazzaro et al. [47]	No	Yes	No	Yes	No	Yes	Yes	Yes	Yes	No	-	-	-	-
Mujica-Mota et al. [48]	No	No	No	Yes	No	Yes	No	Yes	Yes	No	-	-	-	-
Ahola et al. [49]	No	Yes	Yes	No	Yes	Yes	Yes	No	No	No	-	-	-	-
Morelli et al. [50]	No	Yes	No	No	No	No	No	No	No	No	-	-	-	-
Thronicke et al. [51]	No	Yes	Yes	Yes	No	Yes	Yes	No	No	No	-	-	-	

The assessment of the quality of studies provided a picture on common study shortcomings. In particular, for observational studies, information on data collection, time horizon, discount rate and the role of productivity losses was usually lacking in full and partial economic evaluations. For modelling studies, information concerning data identification and incorporation, as well as assessment of external consistency was frequently missing.

**Fig. 2:**
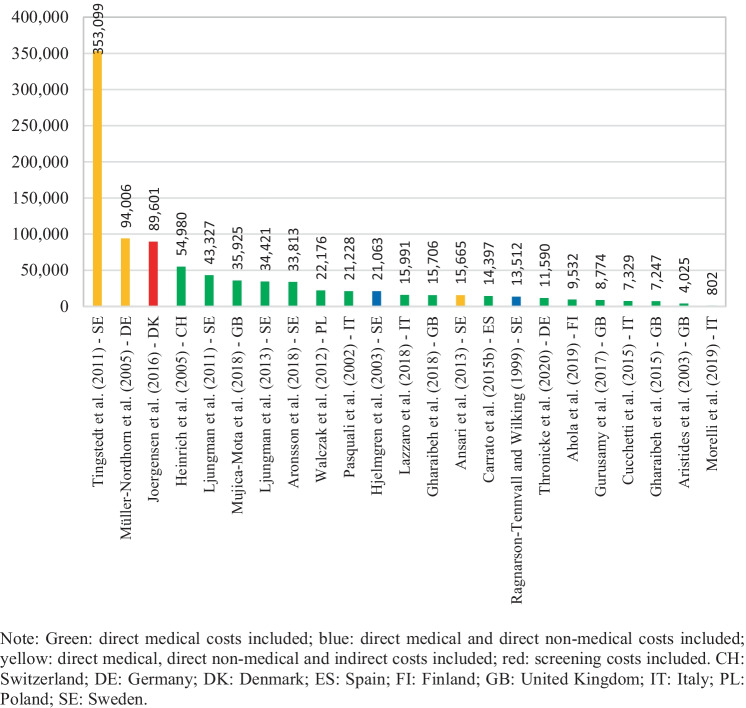
Cost per patient by study

**Fig. 3:**
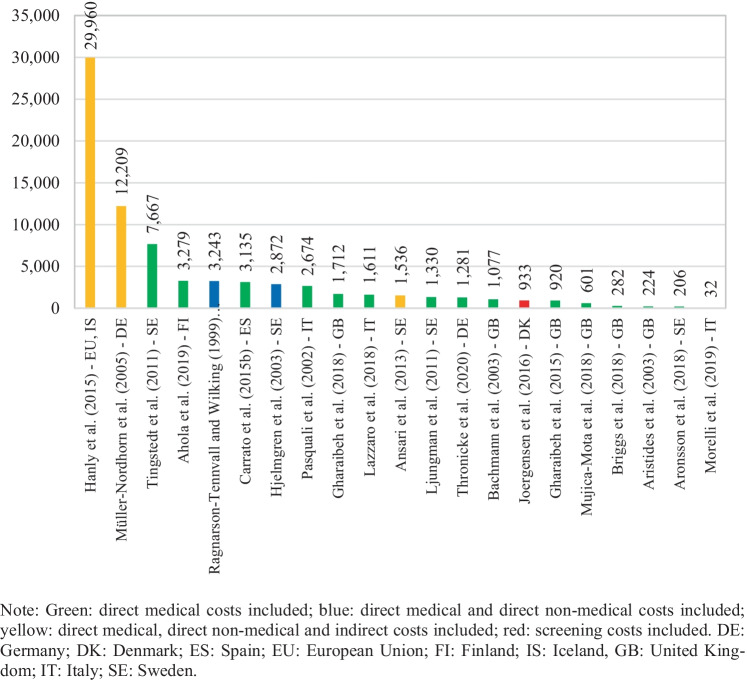
Cost per patient per month by study

Cost per patient information was extracted from 23 of the 26 selected studies. The three missing studies did not include cost information for the whole survival period but rather for a specific unit of time [29, 41, 45]. Costs reported were on average 40,357 euros per patient (median 15,991), and they varied between 353,099 and 802 euros per patient, as seen in Fig. 2. The highest value was found in Tingstedt et al. [34] which estimates treatment costs and productivity losses from a hospital sample in Sweden. The lowest value, cited in Morelli et al. [50], corresponds to surveillance follow-up costs for magnetic resonance imaging (MRI) and ultrasound diagnostics from a hospital sample in Italy. Furthermore, as shown in Fig. 2, highest cost per patient estimations were derived from studies addressing indirect costs in their estimations (marked in yellow) [32, 34] and from a study evaluating the costs of a screening program (marked in red) [42]. Cost per patient figures in Ansari et al. [36] were not among the highest, despite having included indirect costs, most likely because they addressed only those arising from morbidity. In addition, cost PPM information was derived from 21 of the 26 selected studies. Patient survival information was not available in five studies; therefore, monthly costs for these could not be calculated [31, 35, 37, 39, 43]. Cost PPM were on average 3,656 euros across the studies (median 1,536), and they varied between 29,960 and 32 euros, as exhibited in Fig. 3. The highest estimate came from Hanly et al. [41] in which productivity losses from morbidity and mortality are assessed for 30 European countries; the lowest was from Morelli et al. [50].

**Fig. 4:**
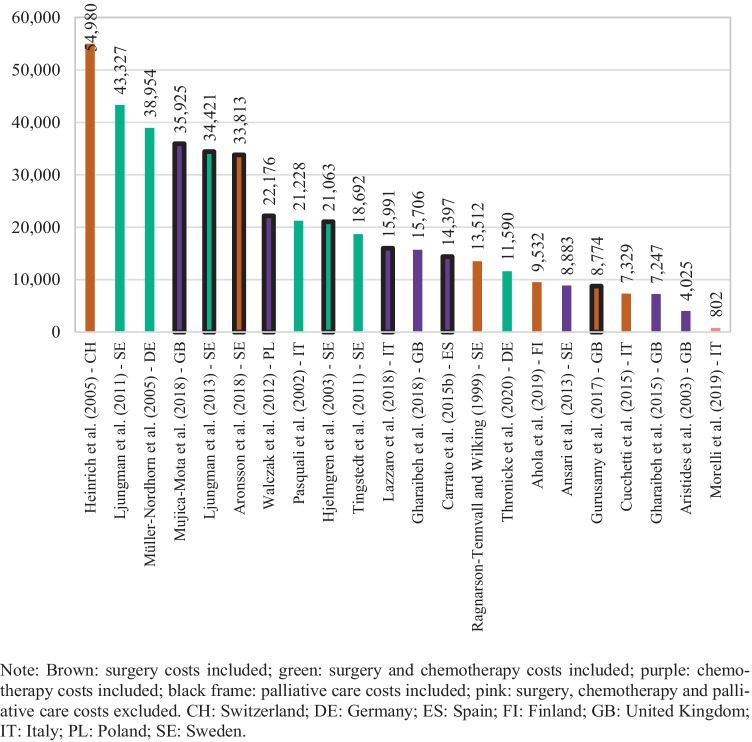
Direct cost per patient by study

**Fig. 5:**
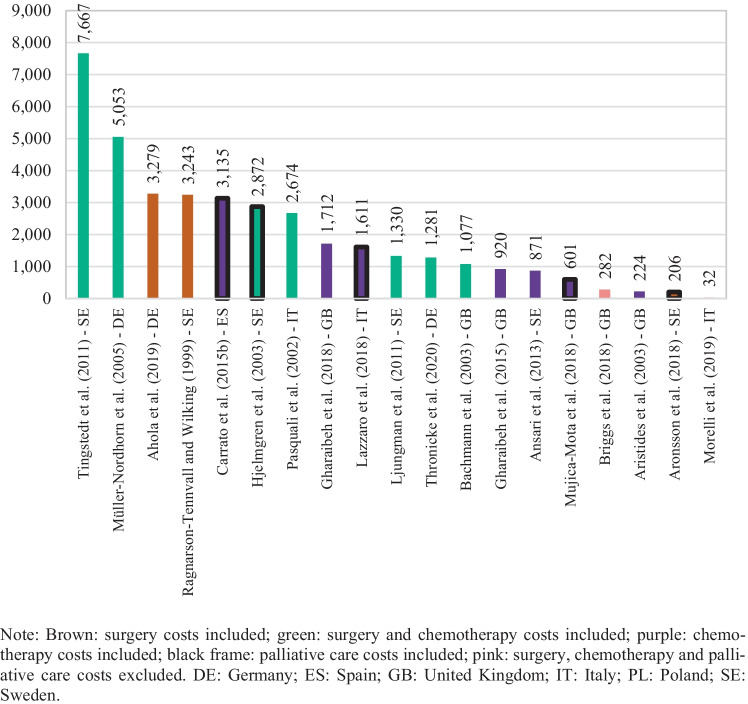
Direct cost per patient per month by study

Figure 4 presents direct costs per patient highlighting the different cost components included in the estimations. From the 23 studies reporting per patient figures, there was one study that did not include direct costs [42]. Average direct costs per patient within these studies were 20,108 euros (median 15,848). Similarly, Fig. 5 shows direct costs PPM for the 19 studies from which such information was retrievable. There were 2 studies, out of the 21 studies with available costs PPM, which did not report on direct costs [41, 42]. Direct costs PPM were on average 2,004 euros (median 1,330). The highest estimate was obtained from Tingstedt et al. [34], in which healthcare resource utilization patterns are followed from diagnosis until death for all identified patients in a hospital sample from Sweden. Müller-Nordhorn et al. [32] used a similar approach with a hospital sample in Germany and revealed the second highest estimate. The lowest values came from Morelli et al. [50]. Moreover, as noted from Fig. 5, higher estimates were derived from studies with therapies comprising of surgery procedures, either in conjunction with chemotherapy (marked in green) or without it (marked in brown). In addition, studies concerning chemotherapy regimens (marked in purple) tended to show lower estimates. There are, however, some exceptions. Aronsson et al. [44] included surgery procedures; yet, direct costs PPM obtained were as low as 206 euros. The model in this study considered patients with branch-duct intraductal papillary mucinous neoplasms that may develop into cancer, and these presented survival times often exceeding 13 years, resulting in low direct costs PPM. Figures per patient were, nevertheless, moderate (Fig. 4). Bachmann et al. [29] and Thronicke et al. [51] also had lower than the median direct costs PPM despite addressing surgery procedures. In the sample by Thronicke et al. [51], however, only a fifth of patients actually underwent surgery. As for Bachmann et al. [29], the patient cohort in the analysis dated from 1996 and treatments guidelines might have differed considerably. On the other hand, direct costs PPM in Carrato et al. [38] were as high as 3,135 euros, although treatment options with only chemotherapy were evaluated. This study, nonetheless, analysed patients in stage IV. The other two studies with above the median direct costs PPM and observing chemotherapy regimens only, namely Gharaibeh et al. [46] and Lazzaro et al. [47], also focused on stage IV patients.

Table 3 presents direct cost per patient by treatment approach from the studies for which this information was presented separately. There were in total seven studies with available estimations for surgery [27, 30, 34, 37, 39, 43, 44], seven for chemotherapy [27, 28, 35, 38, 40, 47, 48] and five for palliative care [35, 37, 39, 44, 48]. Cross study averages were 30,842 euros for surgery, 21,592 euros for chemotherapy and 12,852 euros for palliative care. Cost per patient for surgery are particularly low in Cucchetti et al. [39] and Gurusamy et al. [43], and unlike the other studies, they both consider either mostly or exclusively distal pancreatectomy cases. Among the studies presenting treatment costs for chemotherapy, Aristides et al. [28] did not include costs of disease monitoring, clinical and diagnostic tests, adverse events management or final stage of disease, resulting in relatively low estimates. Cost per patient figures for palliative care in Walczak et al. [35] and Cucchetti et al. [39] were considerably lower than the cross study average, while they considered cases of pancreatic neuroendocrine tumours from an early stage. Furthermore, only two studies, both from Sweden, presented cost per patient by cancer stage [30, 34]. As exhibited in Table 4, cost per patient is highest in the early stage at 26,240 euros on average.

**Table 3:** Direct cost per patient by treatment approach

Surgery			Chemotherapy			Palliative care	
Pasquali et al. [27]	34,117		Pasquali et al. [27]	14,414		Walczak et al. [35]	3,270
Hjelmgren et al. [30]	29,339		Aristides et al. [28]	4,025		Ljungman et al. [37]	26,196
Tingstedt et al. [34]	23,142		Walczak et al. [35]	41,082		Cucchetti et al. [39]	2,876
Ljungman et al. [37]	56,314		Carrato et al. [38]	14,397		Aronsson et al. [44]	15,198
Cucchetti et al. [39]	11,781		Gharaibeh et al. [40]	15,706		Mujica-Mota et al. [48]	16,719
Gurusamy et al. [43]	8,774		Lazzaro et al. [47]	15,991		Average	12,852
Aronsson et al. [44]	52,428		Mujica-Mota et al. [48]	45,528			
Average	30,842		Average	21,592

**Table 4:** Direct cost per patient by cancer stage

	Resectable	Locally advanced	Metastatic
Hjelmgren et al. [30]	29,339	24,501	15,425
Tingstedt et al. [34]	23,142	17,336	18,824
Average	26,240	20,918	17,125

## Discussion

In Europe, cost patient for pancreatic cancer was on average 40,357 euros (median 15,991 euros), while figures PPM were on average 3,656 euros (median 1,536 euros). When a society perspective was adopted and indirect costs were estimated, which was rather uncommon, the cost was on average 154,257 euros per patient or 14,568 euros PPM. In these instances, estimations were considerably higher if indirect costs addressed productivity losses due to mortality instead of those due to morbidity. No study was found in which indirect costs other than productivity losses were evaluated. Whenever only direct costs were considered, cost per patient was 20,108 euros on average (median 15,848 euros) and cost PPM was 2,004 euros on average (median 1,330 euros). This also suggests that indirect costs outweigh direct costs, likely triggered by patients being commonly diagnosed in late stages and consequently having low survival [52]. Direct cost figures did not differ notably by which perspective, that of the payer, service provider, healthcare sector or patient (only one study), was taken. Only a few studies included direct non-medical costs, and these consisted of transportation costs. There is only one study that addressed out of pocket payments.

There are not many studies comprising cost per patient in other cancer sites for different countries in Europe; however, pancreatic cancer costs PPM seem to be in the upper bound relative to other cancer types. McGuire et al. [53], for example, estimated non-small cell lung cancer direct costs PPM to be between 1,316 and 1,600 euros in the first year after the diagnosis for three different countries in Europe in 2012 and between 166 and 1,108 in the second year after the diagnosis. Colorectal cancer direct costs were found in Haug et al. [54] to be 2,162, 191 and 4,307 euros PPM in Germany in 2010, for the initial, intermediate and end of life phases, respectively. On the other hand, Fourcade et al. [55] assessed prostate cancer costs in the first year after diagnosis for five European countries in 2006, obtaining direct costs PPM in a range between 271 and 488 euros. Krensel et al. [56] estimated direct cost for malignant melanoma to average 370 euros PPM for a large of group of countries Europe in 2012. Nevertheless, lifetime direct costs for patients with pancreatic cancer might be lower compared to other cancer types because of shorter survival [6, 8].

Variation across the studies on direct cost estimations was large. These figures were driven by study methodology; patient sample characteristics, such as type of tumour and cancer stage; and the cost components included, such as type of procedure. Cost of illness studies following healthcare utilization patterns in hospitals since diagnosis usually produced larger direct cost per patient and PPM figures than cost-effectiveness studies comparing specific therapies and based on modelling techniques. Studies that included surgery procedures, either in conjunction with chemotherapy or without, resulted in higher direct costs PPM. Here, those addressing tumour types with better prognoses obtained lower PPM figures, but not necessarily different ones per patient. Studies focusing in chemotherapy regimens tended to report lower direct costs per patient and PPM, unless they analysed stage IV patients exclusively. In the latter case, direct costs PPP were around the across study average or above. Although radiotherapy is also recommended in pancreatic treatment guidelines, our review did not identify any study with radiotherapy as main procedure or cost information in this respect.

Only a small number of studies presented cost estimates by treatment approach individually. Surgery costs per patient were found to be 30,842 euros on average across studies with such available information, while chemotherapy costs were 21,592 euros, and palliative care costs were 12,852 euros on average per patient. Surgery costs per patient were lower in studies where surgery cases consisted mostly or exclusively of distal pancreatectomy. Lower chemotherapy costs per patient were obtained from studies addressing fewer cost items in their estimations, such as those for disease monitoring or adverse events management. Studies focusing in neuroendocrine tumours resulted in lower costs per patient for palliate care.

The main limitation of this study is that the large heterogeneity among the articles collected does not allow for comparison across countries. Large contrasts in regard to methodology, patient sample characteristics and cost components included and reported are very likely to drive differences within countries rather than the economic burden of the disease per se. For example, many estimates for Sweden came from cost of illness studies based on patient registry records, which tend to report large figures. In contrast, amounts for the UK were usually retrieved from cost-effectiveness analyses comparing chemotherapy regimens and based on modelling techniques, which deliver relatively lower figures. This might respond to treatment guidelines, in which chemotherapy is the standard of care in advanced stages of pancreatic cancer, when most patients are diagnosed [57–59]. In addition, the small number of existing articles does not permit a rigorous quantitative analysis on the impact of the cost components in total costs.

## Conclusion

Despite its low incidence, pancreatic cancer has a substantial cost on society, mainly as the result of its high mortality rates. Direct costs PPM are in the upper bound relative to other cancer types; however, direct per patient costs are likely to be lower because of shorter survival. Indirect costs will rise in the future as a result of population ageing and increasing retirement ages, translating into larger productivity loses derived from sickness and premature mortality. Existing evidence is, nevertheless, heterogeneous in its objectives and research methods, and generalization is therefore limited. New research should focus in the standard procedures of a typical patient and addressing all possible cost components from a society perspective, instead of specific treatment approaches.

## Data Availability

Publicly available data were used in this review, and details are given in the methodology section. Further information is available from the corresponding author upon reasonable request.

## Appendix 1. Key terms in search algorithm by search engine

**Table:**

Database	Search engine	Filter	Algorithm
Medline	PubMed, Best Match Algo, 27.09.2018, updated version, announced 04.03.2019	01.01.1993 until 31.03.2020, Title/Abstract, Humans	(carcinoma*[Title/Abstract] OR neoplas*[Title/Abstract] OR tumor[Title/Abstract] OR tumors[Title/Abstract] OR cancer*[Title/Abstract]) AND (pancreatic*[Title/Abstract] OR pancreas*[Title/Abstract] OR Pancreatic Diseases[MeSH Terms] OR Pancreatic Neoplasms[MeSH Terms]) AND (cost[Title/Abstract] OR costs[Title/Abstract] OR costing[Title/Abstract] OR economic analys*[Title/Abstract] OR economic evaluat*[Title/Abstract] OR economic loss[Title/Abstract] OR expenditure*[Title/Abstract] OR spend*[Title/Abstract] OR expense*[Title/Abstract] OR burden*[Title/Abstract] OR QALY*[Title/Abstract] OR quality-adjusted life year[Title/Abstract] OR DALY*[Title/Abstract] OR disability adjusted life years[Title/Abstract] OR productivity[Title/Abstract] OR Costs and Cost Analysis [MeSH Terms])
Google Scholar		1993–2020, no patents, no citations, intitle	(intitle:carcinoma OR intitle:carcinomas OR intitle:neoplasm OR intitle:neoplasms OR intitle:tumor OR intitle:tumors OR intitle:cancer OR intitle:cancers) AND (intitle:pancreatic OR intitle:pancreas) AND (intitle:cost)
Google Scholar		1993–2020, no patents, no citations, intitle	(intitle:carcinoma OR intitle:carcinomas OR intitle:neoplasm OR intitle:neoplasms OR intitle:tumor OR intitle:tumors OR intitle:cancer OR intitle:cancers) AND (intitle:pancreatic OR intitle:pancreas) AND (intitle:costs)
Google Scholar		1993–2020, no patents, no citations, intitle	intitle:carcinoma OR intitle:carcinomas OR intitle:neoplasm OR intitle:neoplasms OR intitle:tumor OR intitle:tumors OR intitle:cancer OR intitle:cancers) AND (intitle:pancreatic OR intitle:pancreas) AND (intitle:costing)
Google Scholar		1993–2020, no patents, no citations, intitle	(intitle:carcinoma OR intitle:carcinomas OR intitle:neoplasm OR intitle:neoplasms OR intitle:tumor OR intitle:tumors OR intitle:cancer OR intitle:cancers) AND (intitle:pancreatic OR intitle:pancreas) AND (intitle:economic)
Google Scholar		1993–2020, no patents, no citations, intitle	(intitle:carcinoma OR intitle:carcinomas OR intitle:neoplasm OR intitle:neoplasms OR intitle:tumor OR intitle:tumors OR intitle:cancer OR intitle:cancers) AND (intitle:pancreatic OR intitle:pancreas) AND (intitle:expenditure)
Google Scholar		1993–2020, no patents, no citations, intitle	(intitle:carcinoma OR intitle:carcinomas OR intitle:neoplasm OR intitle:neoplasms OR intitle:tumor OR intitle:tumors OR intitle:cancer OR intitle:cancers) AND (intitle:pancreatic OR intitle:pancreas) AND (intitle:expenditures)
Google Scholar		1993–2020, no patents, no citations, intitle	(intitle:carcinoma OR intitle:carcinomas OR intitle:neoplasm OR intitle:neoplasms OR intitle:tumor OR intitle:tumors OR intitle:cancer OR intitle:cancers) AND (intitle:pancreatic OR intitle:pancreas) AND (intitle:spend)
Google Scholar		1993–2020, no patents, no citations, intitle	(intitle:carcinoma OR intitle:carcinomas OR intitle:neoplasm OR intitle:neoplasms OR intitle:tumor OR intitle:tumors OR intitle:cancer OR intitle:cancers) AND (intitle:pancreatic OR intitle:pancreas) AND (intitle:spends)
Google Scholar		1993–2020, no patents, no citations, intitle	(intitle:carcinoma OR intitle:carcinomas OR intitle:neoplasm OR intitle:neoplasms OR intitle:tumor OR intitle:tumors OR intitle:cancer OR intitle:cancers) AND (intitle:pancreatic OR intitle:pancreas) AND (intitle:expense)
Google Scholar		1993–2020, no patents, no citations, intitle	(intitle:carcinoma OR intitle:carcinomas OR intitle:neoplasm OR intitle:neoplasms OR intitle:tumor OR intitle:tumors OR intitle:cancer OR intitle:cancers) AND (intitle:pancreatic OR intitle:pancreas) AND (intitle:expenses)
Google Scholar		1993–2020, no patents, no citations, intitle	(intitle:carcinoma OR intitle:carcinomas OR intitle:neoplasm OR intitle:neoplasms OR intitle:tumor OR intitle:tumors OR intitle:cancer OR intitle:cancers) AND (intitle:pancreatic OR intitle:pancreas) AND (intitle:burden)
Google Scholar		1993–2020, no patents, no citations, intitle	(intitle:carcinoma OR intitle:carcinomas OR intitle:neoplasm OR intitle:neoplasms OR intitle:tumor OR intitle:tumors OR intitle:cancer OR intitle:cancers) AND (intitle:pancreatic OR intitle:pancreas) AND (“Quality adjusted life years”)
Google Scholar		1993–2020, no patents, no citations, intitle	(intitle:carcinoma OR intitle:carcinomas OR intitle:neoplasm OR intitle:neoplasms OR intitle:tumor OR intitle:tumors OR intitle:cancer OR intitle:cancers) AND (intitle:pancreatic OR intitle:pancreas) AND (intitle:QALY)
Google Scholar		1993–2020, no patents, no citations, intitle	(intitle:carcinoma OR intitle:carcinomas OR intitle:neoplasm OR intitle:neoplasms OR intitle:tumor OR intitle:tumors OR intitle:cancer OR intitle:cancers) AND (intitle:pancreatic OR intitle:pancreas) AND (“disability adjusted life years”)
Google Scholar		1993–2020, no patents, no citations, intitle	(intitle:carcinoma OR intitle:carcinomas OR intitle:neoplasm OR intitle:neoplasms OR intitle:tumor OR intitle:tumors OR intitle:cancer OR intitle:cancers) AND (intitle:pancreatic OR intitle:pancreas) AND (intitle:DALY)
Google Scholar		1993–2020, no patents, no citations, intitle	(intitle:carcinoma OR intitle:carcinomas OR intitle:neoplasm OR intitle:neoplasms OR intitle:tumor OR intitle:tumors OR intitle:cancer OR intitle:cancers) AND (intitle:pancreatic OR intitle:pancreas) AND (intitle:euro)
Google Scholar		1993–2020, no patents, no citations, intitle	(intitle:carcinoma OR intitle:carcinomas OR intitle:neoplasm OR intitle:neoplasms OR intitle:tumor OR intitle:tumors OR intitle:cancer OR intitle:cancers) AND (intitle:pancreatic OR intitle:pancreas) AND (intitle:productivity)
Web of science	v.5.34	1993–2020, all databases, title	(pancrea*) AND (cancer* OR tumor* OR carcinoma* OR neoplasm*) AND (cost* OR spend* OR expen* OR DALY OR QALY OR burden OR euro OR economic OR productivity)
EconLit		January 1993 to March 2020	(pancreas OR pancreatic) AND (cancer* OR tumor* OR carcinoma* OR neoplasm*) AND (cost* OR spend* OR expen* OR DALY OR QALY OR burden OR euro OR economic OR productivity)
